# New Elements of Operative Surgery

**Published:** 1847-07

**Authors:** 


### BIBLIOGRAPHICAL NOTICES.

---

ART. IV.—*New Elements of Operative Surgery.* By ALPH. A. L. M. VELPEAU. Translated by P. S. TOWNSEND, M.D., late Physician to the Seamen's Retreat, Staten Island, New York, *under the supervision of, and with Notes and Observations by,* VALENTINE MOTT, M.D., Professor of Operative Surgery in the University of New York. Vol. III: with an Atlas. New York: Samuel S. and William Wood: 1847.

We are indebted to the new publishers for the concluding parts of this great work, consisting of the third volume and an Atlas. The translator takes occasion in this volume to sneer at what he terms the "professed and maudlin criticisms" of his labors which have appeared in "sundry" journals. We do not at present propose to extend the number of these criticisms, but we hope at an early day to find leisure to enter upon a thorough examination of these volumes as they now stand before the American profession. Taken as a whole, the work must be pronounced an extraordinary one. The labor of producing it, unquestionably, was immense; it exhibits research and learning far beyond what is often met with in this our day of rapid writing, and on this account has high claims upon the student and practitioner of surgery. But when we turn to the contributions which it has received on this side of the Atlantic, we find a mass of matter, possessing intrinsic interest indeed, but *ruda indigestaque* to the last degree; so that we do not see how the work can escape criticism. Possessing uncommon merits, it is marred by blemishes which occur to the most superficial reader; but these, let us add, are of a nature to be corrected by patient industry and more enlarged experience; and we therefore recommend to the translator to exercise a little more charity towards his critics, and profit by their admonitions.

